# Effect of Decontamination Treatments on Micro-Shear Bond Strength between Blood–Saliva-Contaminated Post-Etched Dentin Substrate and Composite Resin

**DOI:** 10.3390/healthcare7040128

**Published:** 2019-11-01

**Authors:** Satheesh B. Haralur, Salem Mohammed Alharthi, Saeed Aied Abohasel, Khalid Mohammed alqahtani

**Affiliations:** Department of Prosthodontics, College of Dentistry, King Khalid University, Abha 62529, Saudi Arabia; S.m.a1414al@hotmail.com (S.M.A.); Saeed77b@yahoo.com (S.A.A.);

**Keywords:** dentin bonding agents, blood–saliva contamination, micro-shear bond strength, dentin decontamination method

## Abstract

Blood–saliva contamination negatively affects the bonding potential of adhesive agents. The study aimed to assess the effect of various cleaning protocols on micro-shear bond strength (μSBS) between blood–saliva-contaminated post-etched dentin and composite resin in total-etch and self-etch adhesives. The cleaning methods tested were water rinsing, 37.5% phosphoric acid (H3PO4) re-etching, 6% sodium hypochlorite (NaoCl), 2% chlorhexidine gluconate (CXG), isopropyl alcohol (IPA), and pumice. Nono-hybrid composite cylinders with a 3-mm diameter and 2-mm height were directly cured over the dentin substrate, stored for 24 h, and subjected to 12,000 thermocycles. The shear force was exerted with a 200-μm knife-edged chisel-shaped head from a universal testing machine. The type of failure was assessed with stereomicroscope magnified images. The obtained data were evaluated by Kruskal–Wallis and Mann–Whitney U post-hoc tests. Water-rinsed contaminated dentin surfaces showed substantially reduced μSBS in the total etch from 25.93 to 20.29 Mpa and the corresponding values for the one-step self-etch adhesive were 10.10 to 8.8. Re-etching with 37.5% H3Po4 resulted in a recovery of bonding potential in both total-etch (24.58 Mpa) and self-etch adhesive (9.23 Mpa). Alternately, NaoCl and pumice cleaning showed promising results for the total-etch (23.51 Mpa) and self-etch (7.79 Mpa).

## 1. Introduction

The demand for tooth-colored restoration is constantly increasing because of the image-focused modern society. Utilization of both direct and indirect aesthetic restorations like composite and ceramics with invisible margins and insistence for high aesthetic outcomes is on the constant upsurge in contemporary dental practice [1]. Composite restorations are extensively utilized to restore a fractured or carious tooth using a minimally invasive approach. Buonocore initially proposed the bonding of resin [2] with his acid etch technique; it was presumed to facilitate micromechanical retention. However, recent studies have concluded that successful bonding between the tooth substrate and restorations is a combined outcome of micromechanical retention and chemical bonding by functionalized monomers with hydroxyapatite [3]. The optimum bonding strength between the restoration and tooth structure is crucial for long clinical service. Inadequate bonding and the existence of a micro gap between the restoration and tooth structure leads to discoloration, secondary caries, postoperative hypersensitivity, and subsequent failure of the restoration. The clinical procedure to achieve effective and durable bonding between the tooth substrate and dentin bonding agents is highly technique sensitive. Meticulous tooth surface preparation, conditioning, and control of the environment is critical for successful bonding.

Contamination of the prepared tooth surface from saliva, blood, water, and gingival sulcular fluid potentially compromises the bonding strength of dentin bonding agents [4,5]. The prevention of fluid contamination of the prepared tooth surface is challenging in many clinical situations. Clinical situations like non-viability for rubber dam applications, sialorrhea patients, surgically operated areas, and deep proximal restorations predispose the prepared tooth surface for fluid contamination. Blood protein contents along with fibrinogen and platelets form a layer over the dentin surface, thus preventing the penetration of an adhesive within the dentinal tubules [6]. Protein components of blood also react with dentin collagen networks, leading to interference in chemical bonding to the tooth substrate. Routinely, clinicians adopt either of two strategies to decontaminate the tooth surface. The first one is repeating the adhesive procedure or applying different cleaning agents to neutralize the effects of blood and saliva. The decontamination protocols recommended by earlier researchers are dissimilar. Dias Damé JL [7] concluded that water rinsing is a reliable procedure for cavity decontamination. Dina Elkassas et al. [8] recommended re-etching rebonding of the affected dentin surface while Juneja R et al. [9] suggested the application of NaOCl for the recovery of the bonding potential on the contaminated tooth surface. Few researchers suggest resurfacing with a rotary instrument, followed by water irrigation and reapplication of an adhesive system [10]. Park et al. [11] proposed the blot drying of saliva from an etched dentin surface to regain the bond strength. Lee SB et al. [12] recommended the utilization of catechol-functionalized synthetic polymer as a dental adhesive to the contaminated dentin surface.

Modern dentin bonding strategies mainly comprise the total-etch and one-step self-etch adhesive techniques. The total-etch technique incorporates the superficial dissolution of dentin and enamel by phosphoric acid. Subsequently, polymerizable monomers condition the modified dental hard tissues. The main advantage of the total-etch technique is its high bond strength; its limitations include post-operative sensitivity, nono-leakage, and technique-sensitive procedures. Researchers, in an effort to improve the bonding performance, reliability, and simplification of the clinical procedure, developed a one-step self-etch bonding technique. Self-etching primers include the methacrylate phosphoric acid ester molecule composed of phosphoric acid and a methacrylate group. During the application of one-step self-etch adhesives, the surface is simultaneously infiltrated with adhesive along with surface etching by acidic monomers. There is a significant difference in the clinical procedure, component, and bonding chemistry between the etch-wash and self-etch bonding techniques. Hence, the effect of contamination and decontamination methods on the tooth surface is expected to be different [13].

The adhesive stage at which the tooth surface is contaminated is also a critical factor in assessing the extent of damage to the bonding potential. The contamination of a prepared tooth surface after etching is a common occurrence in clinical practice. The force of water irrigation, the corrosive effect of the acid etchant, and inflamed adjacent soft tissues predispose the surface to contamination with blood and saliva. Though the cleaning protocols of blood- and saliva-contaminated dentin surface have been extensively studied, recommendations from earlier researchers are contradicting and diverse. Studies regarding the effective cleaning protocol for both total-etch and self-etch adhesive systems are scarce. There is a need for studies to further the evidence of the effectiveness of these decontamination methods for the post-etched dentin surface. In clinical practice, fluid contamination during the restorative procedure includes a mixture of both blood and saliva. The effective sanitization methods for dentin surfaces contaminated with a blood and saliva mixture are clinically more relevant. The study results would assist clinicians in choosing an appropriate decontamination method specific to the bonding strategy used. This in-vitro study assessed the hypothesis that various decontamination protocols are effective treatment options to recover the bonding strength between the composite restoration and fluid-contaminated dentin substrate. The study aimed to investigate the effect of different tooth surface cleaning protocols on the micro-shear bond strength between blood–saliva contaminated tooth surface and composite in etch-wash and self-etch bonding systems.

## 2. Materials and Methods

Intuitional ethical review board authorization was obtained for the study proposal (SRC/ETH/2018-19/008). A total of 75 intact third molar teeth were collected from oral surgery clinics. Consent from the patients was obtained to use their extracted teeth for research purposes. The teeth were extracted for therapeutic purposes and were devoid of caries and restorations. The soft periodontal tissue was cleaned with an ultrasonic scaler, stored in a 10% aqueous formalin solution at room temperature.

**Figure 1:**
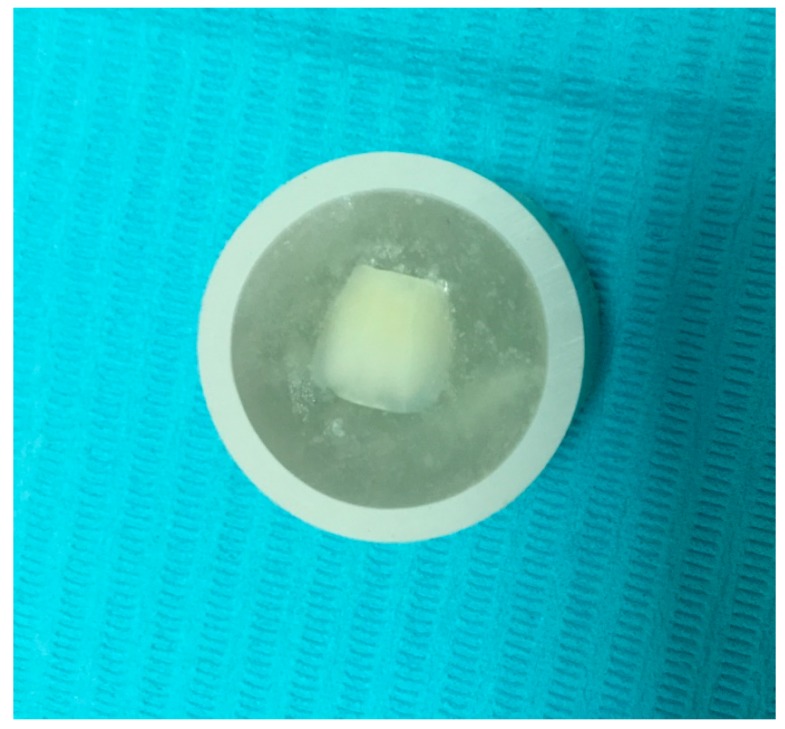
Embedded teeth sample in acrylic resin.

**Figure 2:**
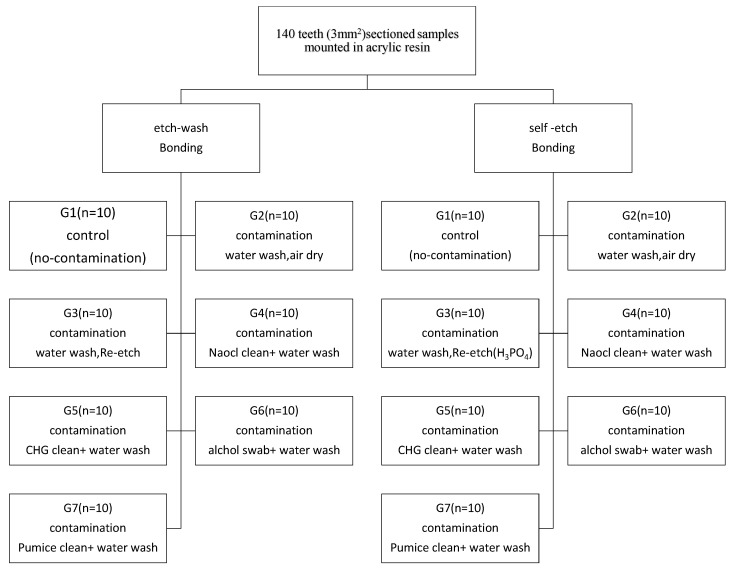
Flowchart of the method: Sample distribution.

The root and cusps of teeth samples were removed by sectioning them at the cementum–enamel junction and depth of the central fossae. They were further segmented vertically into buccal and lingual halves with the help of a double-sided diamond disc (Kerr Corporation, Orange, CA, USA) under a water coolant. Each vertically segmented half was embedded within the acrylic resin. During the implanting procedure, the buccal/lingual surface of the sample was kept parallel to the outer surface with the help of a vertical holding machine. The lower dentin substrate was exposed by grinding the implanted tooth surface for 2 mm with a 400-grit SiC paper disc. A 3-mm^2^ flat dentin area was achieved by additional grinding with a 600-grit SiC paper disc (Figure 1). The prepared samples were randomly divided into two major groups to be bonded with total-etch and self-etching adhesive protocols. Each adhesive system was further subdivided into 7 groups (*n* = 10). In total, 10 specimens per testing group were estimated according to previously published studies [14,15]. The sample size was calculated with G* Power software (version 3.1; University of Dusseldorf), with an effect size (d) of 1.4, *α* of 0.05, and 1-*β* (power) of 0.85 [16]. The effect size was calculated from the bonding strength values of blood/saliva-contaminated to non-contaminated dentin substrate from earlier studies [14,15]. A flow chart of the sample distribution is shown in Figure 2. Both unstimulated saliva and fresh blood from the needle-punctured fingertip were collected from the single investigator. Capillary blood is recommended by earlier researchers [17] instead of heparinized blood. Hence, fresh blood was used in this study. The fluid used to contaminate the dentin surface was comprised of blood and saliva in equal proportions. A description of the surface treatment and bonding procedure for each subgroup is given in Table 1.

**Figure 3:**
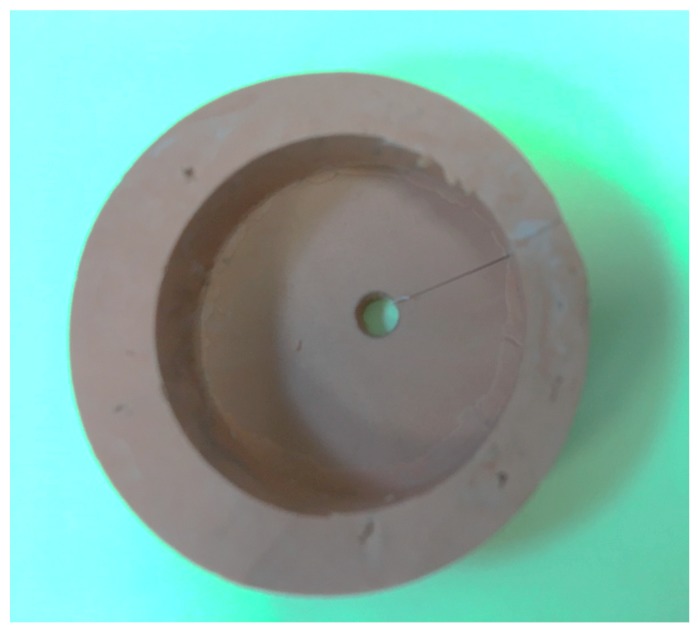
Custom-made additional silicone mold to standardize the composite cylinder size and location.

**Figure 4:**
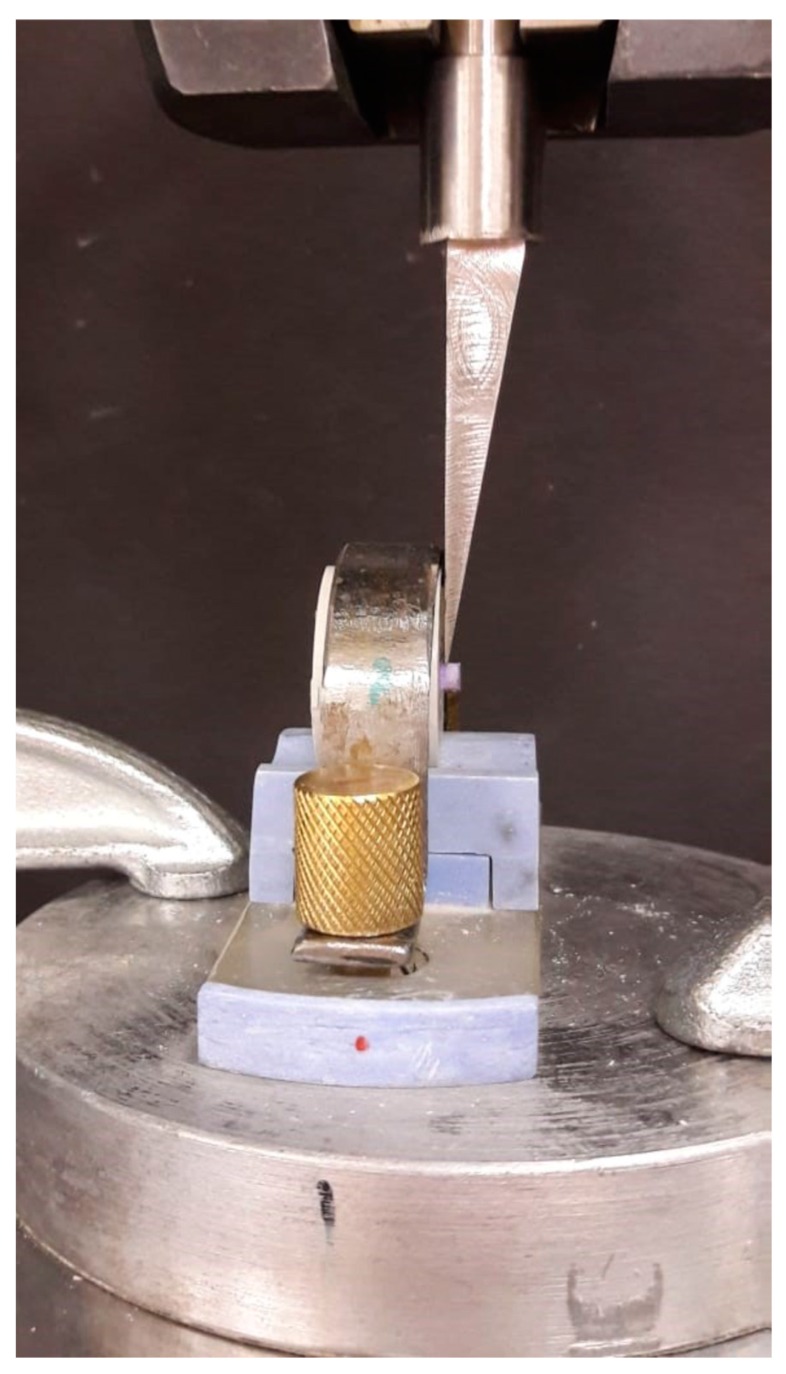
Macro shear bond strength evaluation with INSTRON.

The nono-hybrid composite cylinder (Tetric N-Ceram, Ivoclar Vivodent AG, Schaan, Liechtenstein) with a diameter of 3 mm and height of 2 mm was polymerized on to the flat dentin surface according to the manufacturer’s instructions. To standardize the composite cylinder, a custom-made additional silicone putty mold was made with a cylindrical space at the center corresponding to the embedded teeth sample (Figure 3). The composite resin was packed and covered with a glass slab to prevent exposure to oxygen, and to get a flat top surface. Composite resin was cured for 20 s with a light-emitting diode light-curing unit (Bluephase; Ivoclar Vivadent) at 700 mW/cm^2^. After removal of the mold, the composite cylinders were light-cured for an additional 20 s. The shear bond strength of samples was tested according to the ISO/TS 11405:2015 specification. The bonded restorations were stored in distilled water for 24 h at 37 °C. Subsequently, the specimens were subjected to 12,000 thermal cyclings (Thermocycler, SD Mechatronik, Feldkirchen-Westerham Germany) between 5 and 55 °C with a 30-s dwelling time. The shear force was exerted with a 200-μM chisel-shaped head at a crosshead speed of 1 mm/min with a universal testing machine (Instron, Norwood, MA, USA) (Figure 4). Shear bond stress at maximum load is expressed in Mpa. The de-bonded interfaces were assessed with a stereomicroscope ×25 (Olympus/DeTrey, Germany) to determine the failure modes. Statistical analysis was performed using the SPSS 19 software (IBM Corporation, Armonk, NY, USA). The data were evaluated by Kruskal–Wallis and Mann–Whitney U post-hoc tests. The level of statistical significance was determined at *p* < 0.05.

## 3. Results 

The results (Table 2) confirm the deleterious effect of the blood/saliva-contaminated teeth surface on the composite bond strength. The resultant reduction of μSBS due to contamination in total etch was from 25.93 to 20.29 Mpa, and the corresponding values for self-etch were 10.10 to 8.83 Mpa. Among the surface treatments tested in the total-etch bonding technique, re-etch was most efficient, with an μSBS of 24.58 Mpa, followed by NaoCl (23.25 Mpa). The cleaning by CHG (14.95 Mpa), alcohol swab (13.14 Mpa), and pumice (9.73) were found to be ineffective. Regarding the self-etch bonding system, re-etch recorded a better μSBS, with 9.23 Mpa, followed by pumice (7.79 Mpa), NaoCl (7.20 Mpa), and CHG (6.58 Mpa).

Kruskal–Wallis analysis (Table 3) showed a statistically significant difference in μSBS between the different types of surface treatment protocols in the total-etch bonding technique (H (6) = 65.76, *p* = 0.001). The mean rank for the non-contaminated surface was 55.30. Re-etch recorded the highest mean rank for decontamination protocols at 55.95, followed by NaoCl cleaning (46.95), water wash (35.60), and CHG (25.00). The lower mean rank of 5.50 was observed in the group that used pumice cleaning. The Mann–Whitney post-hoc pair-wise comparison for total-etch (Table 4) showed significant differences between the groups for non-contamination and wash (*p* < 0.05, r = −0.84), re-etch (*p* < 0.01, r = −0.54), NaOCl (*p* < 0.05, r = −0.81), CHG (*p* < 0.05, r = −0.84), IPA (*p* < 0.05, r = −0.84), and pumice (*p* < 0.05, r = −0.84). The analysis showed no statistically significant difference between the groups regarding wash vs. re-etch (*p* = 0.199), NaOCl vs. pumice (*p* = 0.173), and CHG vs. isopropyl alcohol (*p* = 0.273).

The self-etch adhesive (Table 3) groups also showed a statistically significant μSBS (H (6) = 54.33, *p* = 0.001). A post-hoc test using Mann–Whitney tests (Table 5) showed significant differences between the groups of non-contamination and wash (*p* < 0.05, r = −0.65), re-etch (p < 0.01, r = −0.50), NaOCl (*p* < 0.05, r = −0.81), CHG (*p* < 0.05, r = −0.84), IPA (*p* < 0.05, r = −0.84), and pumice (*p* < 0.05, r = −0.82). The analysis showed no statistically significant difference between the groups except between wash vs. re-etch (*p* = 0.199), NaOCl vs. pumice (*p* = 0.173), and CHG vs. isopropyl alcohol (*p* = 0.273).

The uncontaminated total-etch adhesive groups showed predominantly mixed and cohesive failure (dentin) while most failures in one-step etch were also mixed, and cohesive at the composite resin (Table 6). A higher frequency of adhesive failures in total-etch was recorded in the pumice and IPA groups. However, among the self-etching groups, higher adhesive failures were observed in the IPA and CHG groups.

## 4. Discussion

Composite resin restorations require a completely isolated tooth substrate to achieve an optimum bond strength. In clinical practice, because of many clinical situations, isolating the working area is challenging. Contamination can occur before or after the etch bonding procedure. Frequently, clinicians encounter blood and saliva contamination after the etching priming procedure. To further existing knowledge, we used the μSBS testing protocol to analyze the effectiveness of cleaning methods in regaining the bonding strength in blood- and saliva-contaminated post-etched primed dentin substrate. The components and development of bonding in total-etch and self-etch adhesive strategies are significantly different. Hence, it is prudent to test cleaning protocols in both adhesive strategies.

Our study reports showed the total-etch bonding system had a higher μSBS strength compared to one-step self-etch adhesives. This is supported the results of earlier researchers, such as Torres et al. [18] and Dos Santos, R.A et al. [19]. Solvent properties, like vaporization speed, drying patterns, and penetration properties, have been reported to influence the bond strength. The μSBS difference between the groups could also be due to the dissimilarity of the hybrid layer structure. Lateral branches within hybrid layers are observed in total-etch while researchers report water droplets within an all-in-one self-etch adhesive layer because of water osmosis from dentin [20]. Moreover, incomplete polymerization of the monomer is also credited for a lesser bonding strength in a water-based single-step self-etch bonding system. Ethanol and acetone solvent-based dentin bonding agents react better with the dentin substrate because of the high volatility in contrast to water [21].

We observed a significant reduction in μSBS in both total-etch and self-etch bonding techniques because of blood and saliva contamination. Dentin comprises organic collagen fibers and 68% inorganic hydroxylapatite. The bonding mechanism in dentin predominantly depends on hybridization and infiltration of resin within the exposed collagen fiber network [22]. The acid-etching procedure exposes the collagen matrix. It is occluded with blood protein, and consequently, hinders the penetration of primer- adhesive components within the collagen network [23]. Over-wetting of the dentin surface, adsorption of salivary glycoproteins on the bonded surface, and diffusion of high-molecular weight macromolecules within dentinal tubules are inferred in the reduction of bond strength in salivary contamination [24]. Saliva and blood contamination are known to affect the bonding strength differently due to the dilute nature of saliva; it is comprised of over 99% water [25]. Earlier reports on the effect of saliva on SBS are contradictory. Korkmaz Sayinsu [26] suggested that blood contamination on acid-etched surfaces affects bond strength more than salivary contamination. CharuphanOonsombat et al. [27] reported a significant reduction of μSBS from both blood and salivary contamination in a self-etch bonding system. Some researchers have reported the insignificant effect of salivary contamination on bonding strength [28]. Total-etch adhesives were affected more than the self-etch groups in our study; this is in agreement with earlier reports from Kermanshah et al. [29]. The one-step self-etch adhesive may be affected to a lesser extent due to the water-based primer, and its hydrophilicity may allow diffusion through the blood and saliva layer. Moreover, our study results showed that the post-primer and adhesive contamination in the self-etch group also led to a reduction of μSBS. This could be because of the absorption of glycoprotein to the incompletely polymerized adhesive surface and inhibition of copolymerization on the resin layer [30].

Our study results confirmed the observation of Soares et al. and Raffaini et al. of an inadequate recovery of bonding strength when utilizing simple water rinsing [31,32]. Chang et al. are of the opinion that water rinsing is ineffective due to higher blood protein molecules resisting the rinsing and inhibiting adhesive infiltration [33]. The decontamination method found to be effective in restoring the bond strength in both adhesive strategies was re-etching the dentin substrate with 37% phosphoric acid for 10 s. Re-etching results in acid denaturation of organic fragments within a collagen network, thus reducing its affinity to the substrate and is easily able to be washed [34]. The re-etching of the contaminated surfaces in the self-etch surface also recorded an improvement in the bonding strength from 8.83 to 9.23 Mpa. The results are in agreement with Furuse et al. [35], who reported that the re-etching of a cured adhesive eliminated contaminated residue and removed the adhesive coating. The re-bonding after a re-etching procedure assists in the refurbishing of patent adhesives for bonding [8].

The study suggests a substantial recovery in bonding strength in both the total-etch and self-etch groups with NaoCl application. An improved bonding potential with NaoCl is credited to its proteolytic action, enabling the removal of organic debris [36,37]. The application of NaoCL is also reported to enhance valley fluid retention and facilitate resin tag formation within the smear layer [38]. Though complete recovery of bonding strength with NaoCl was not achieved, this could be due to the inhibition of polymerization considering its strong oxidizing nature [39]. The results are in agreement with the earlier reports of recommending re-etching and NaoCl cleaning to regain the bonding strength of contaminated dentin substrate post-etching [9]. The 2% chlorhexidine gluconate disinfection was ineffective in restoring the bonding strength in both total-etch and self-etch bonding protocols. CHG is widely recommended to reduce the potential of residual caries and to preserve dentin bonding strength due to its matrix metalloproteinase inhibitor property. Matrix metalloproteinase (MMP) is partially responsible for hybrid layer degradation. The reduced μSBS could be due to the reaction between CHG and phosphate ions from the tooth substrate, leading to microscopically visible precipitates on the dentin surface. This layer alters tooth substrate morphology and hinders the bonding mechanism [40]. CHG appears to encourage calcium ionic dissociation from the dentin surface and seems to lixiviate upon air-drying, resulting in reduced Ca^+2^ availability for chemical bonding. Our results are in agreement with earlier studies reporting an adverse effect of CHG on bonding efficacy [41]. Contrary outcomes of improved bond strength [38] and non-influence from CHG have also been reported by a few researchers [42]. The dissimilarity of outcomes could result from the variation in time of CHG application before vs. after etching, and water rinsing after CHG application. The cleaning of the contaminated surface by isopropyl alcohol swabs was found to be ineffective in both the total-etch and one step self-etch system. This could be because of the precipitation of blood protein components [43]. Among the total-etch decontamination protocols, a larger negative effect size of −0.84 was recorded by the CHG, IPA, and pumice groups. However, the re-etch groups recorded a medium negative effect size of −0.54 and −0.50 for the total-etch and self-etch groups, respectively. This indicated the CHG, IPA, and pumice treatment substantially decreased the mean SBS compared to the non-contaminated dentine surface in the total-etch groups. However, re-etch could revive the SBS of contaminated dentin surfaces to a larger extent in both bonding protocols. The results are similar to earlier reports from Furuse et al. [35], Fawzy AS [37], and Morris MD et al. [38].

Pumice is routinely utilized for dental plaque removal and to clean the provisional cement. Our study results showed significantly less success in restoring the bonding potential in the total-etch group while it performed better in the self-etch group. The exposed collagen network in the total-etch technique is more susceptible to pumice ingress compared to one-step self-etch bonding. As the etching and infiltration of adhesive in one-step self-adhesive are simultaneous, the access for pumice intrusion is limited. The results validate the reports from Sarac et al. [44], who concluded after SEM observation that the force from the rotary instrument during pumice application plugs the dentinal tubule and reduces the surface area for micro-mechanical interlocking. The enhanced bonding potential in the self-etch group could be due to the formation of a fresh dentin substrate by the abrasive action of the pumice. The results are in corroboration with the findings of Al-Twaijri et al. [45]. The total-etch technique is comprised of an acid etchant with low pH and separate etching and bonding procedures. This could lead to a discrepancy in the extent of etching and adhesive infiltration. The deep layers could not be completely bonded with adhesive, resulting in an area of least resistance. The one-step self-adhesive techniques include simultaneous dentin demineralization and adhesive resin infiltration. Hence, inconsistency of failure modes in both adhesive techniques is related to the bonded interface structure.

Taking into account the higher bond strength after 10 s of re-etching with H3PO4 in both the total-etch and self-etch adhesive, we recommend it as a preferred cleaning protocol for blood- and saliva-contaminated dentin substrate. Alternatively, we propose NaoCl for total-etch and pumice cleaning for one-step self-adhesives to restore the bonding potential in blood- and saliva-contaminated dentin surfaces. One of the limitations of the study is that the micro-shear bond strength testing protocol was utilized for testing the bond strength. The influence of viscoelastic properties of the composite resin is not accounted for in this testing method; hence, authors suggest validating the results in the future with alternative testing methods like fracture toughness or fracture mechanics. Though the re-etching of dentin showed a revival of the bonding potential to a larger extent, this procedure may lead to increased interfacial micro-leakage. Hence, it is suggested that the effect of re-etching on micro-leakage values is tested.

## 5. Conclusions

Within the limitation of this in-vitro study, 37.5% H_3_PO_4_ re-etching was effective in restoring the bonding strength of blood- and saliva-contaminated post-etched dentin substrate for both total-etch and self-etch adhesive. NaoCl was found to be effective in cleaning the dentin substrate in the total-etch adhesive while cleaning with non-fluoridated pumice was more appropriate for self-etch adhesive.

## Figures and Tables

**Table 1:** Description of the surface treatment and bonding procedure for each subgroup as follows.

Group	Total-Etch	Self-Etch
No contamination	37.5% phosphoric acid gel (Kerr, Karlsruhe, Germany) 15 s. Water rinse 10 s, gentle air dry. Apply bonding agent (Tetric-N Bond total etch, Ivoclar Vivadent AG, Schaan, Liechtenstein). Brush the material gently into the dentin for 10 s, air dry and light cure 10 s (LED unit (700 mW/cm^2^)	Applied one layer of one bottle self-etch bonding agent (G-ænial, GC America Inc. St, Alsip, IL, USA) left undisturbed 10 s, dry thoroughly for 5 s, light cure 10 s with LED unit (700 mW/cm^2^)
Contamination water rinse	Contaminated the etched surface with blood/saliva mix for 1 min, water rinsed for 10 s, gently air dried for 5 s, application of bonding agent, composite application	Contaminated the self-etched surface with blood/saliva mix for 1 min, water rinsed for 10 s, Gently air dried for 5 s. Re-application of adhesive
Contamination, water wash, Re-etch	Blood/saliva contamination of etched surface, water rinsed, air dried, subsequently re-etched with 37.5% H_3_PO_4_ for 10 s, application of bonding agent	Contaminated the self-etched surface with blood/saliva mix, water rinsed, air dried, re-etched with 37.5% H_3_PO_4_ for 10 s, application of self-etch bonding agent
Contamination Naocl clean, water wash	Blood/saliva contamination of etched surface, 6% sodium hypochlorite (Vita dental products, Racine, WI, USA) applied with micro brush for 15 s, water rinse for 10 s, application of bonding agent	Contaminated the self-etched surface with blood/saliva mix, 6% sodium hypochlorite applied with micro brush for 15 s, water rinse for 10 s, re-apply self-etch bonding
Contamination, water wash CHG disinfection	Blood/saliva contamination of etched surface, water rinse for 10 s, 2% chlorhexidine gluconate (Consepsis, Ultradent INC, South Jordan, UT, USA) rub the area for 10 s, air dried and bonding agent application	Contaminated the self-etched surface with blood/saliva mix, water rinse for 10 s, 2% chlorhexidine gluconate (Consepsis, Ultradent INC, South Jordan, UT, USA) rub the area for 10 s, air dried and re-apply self-etch bonding
Contamination, water wash, isopropyl alcohol swabs	Blood/saliva contamination of etched surface, water rinse for 10 s, rub the surface with 70% isopropyl alcohol swabs (sterie swab), for 10 s, bonding agent application	Contaminated the self-etched surface with blood/saliva mix, water rinse for 10 s, rub the surface with isopropyl alcohol swabs for 10 s, re-apply self–etch bonding
Contamination, cleaning with pumice, alcohol swabs	Blood/saliva contamination of etched surface, water rinse, rub the area with un-fluoridated pumice for 15 s at 2000 rpm prophy brush, water rinse, bond agent application	Blood/saliva contamination of etched surface, water rinse, rub the area with un-fluoridated pumice for 15 g, water rinse, re-apply self-etch bonding

**Table 2:** Descriptive statistics of the Shear bond strength (Mpa) measured in the different groups.

Group	*N*	Total-Etch	Self-Etch
		Mean (SD)	Mean (SD)
No Contamination	10	25.93 (0.96)	10.10 (0.59)
Wash	10	20.29 (1.08)	8.83 (0.81)
Re-Etch	10	24.58 (0.94)	9.23 (0.83)
NaOCl	10	23.25 (1.08)	7.20 (0.56)
CHG	10	14.95 (0.80)	6.58 (0.45)
IPA	10	13.14 (0.86)	6.25 (0.66)
Pumice	10	9.73 (0.83)	7.79 (0.97)

NaOCl = sodium hypochlorite, CHG = chlorhexidine gluconate, IPA = isopropyl alcohol.

**Table 3:** Kruskal–Wallis analysis on SBS measured on different groups.

Surface Treatment	Total-Etch Bonding				Self-Etch Bonding			
	Mean Rank	χ^2^	df	*p*	Mean Rank	χ^2^	df	*p*
No Contamn	63.50	65.768	6	0.000	62.30	54.33	6	0.000
Wash	35.60				48.25			
Re-Etch	55.95				52.30			
NaOCl	46.95				26.25			
CHG	25.00				15.35			
IPA	16.00				11.25			
Pumice	5.50				32.80			

χ^2^ = chi-square, significance level is 0.05.

**Table 4:** Mann–Whitney post-hoc comparison between the SBS recorded in total-etch groups.

Group	Wash	Re-Etch	NaOCl	CHG	IPA	Pumice
No Contamn	0.000	0.016	0.000	0.000	0.000	0.000
Wash		0.000	0.000	0.000	0.000	0.000
Re-Etch			0.006	0.000	0.000	0.000
NaOCl				0.000	0.000	0.000
CHG					0.001	0.000
IPA						0.000

Significance *p* ≤ 0.05.

**Table 5:** Mann–Whitney post-hoc comparison between the SBS recorded in self-etch groups.

Group	Wash	Re-Etch	NaOCl	CHG	IPA	Pumice
No Contamn	0.003	0.023	0.000	0.000	0.000	0.000
Wash		0.199	0.000	0.000	0.000	0.007
Re-Etch			0.000	0.000	0.000	0.010
NaOCl				0.021	0.002	0.173
CHG					0.273	0.007
IPA						0.004

Significance *p* ≤ 0.05.

**Table 6:** Fracture mode recorded for each group (in number).

Group	Total-Etch			One-Step Self-Etch		
	Adhesive	Cohesive	Mixed	Adhesive	Cohesive	Mixed
No contamination	0	3	7	1	3	6
Water Irrigation	4	2	4	3	2	5
Re-etch (37% H_3_PO_4_)	1	3	6	2	2	6
NaOCl	3	1	6	2	1	7
CHG	4	1	5	4	2	4
IPA	5	1	4	6	0	4
Pumice	6	0	4	2	2	6

## Data Availability

The data used to support the findings of this study are available from the corresponding author upon request.
